# Supplementary material for: Cloaking nanoparticles with protein corona shield for targeted drug delivery
Source: Nat Commun. 2018 Oct 31;9:4548. doi: 10.1038/s41467-018-06979-4 (PMC6208370; doi:10.1038/s41467-018-06979-4)
Supplement: Supplementary file 1 — Supplementary Information [file 41467_2018_6979_MOESM1_ESM.pdf]

Supplementary Information

Cloaking nanoparticles with protein corona shield for targeted drug delivery

Oh et al.

Supplementary Figures.

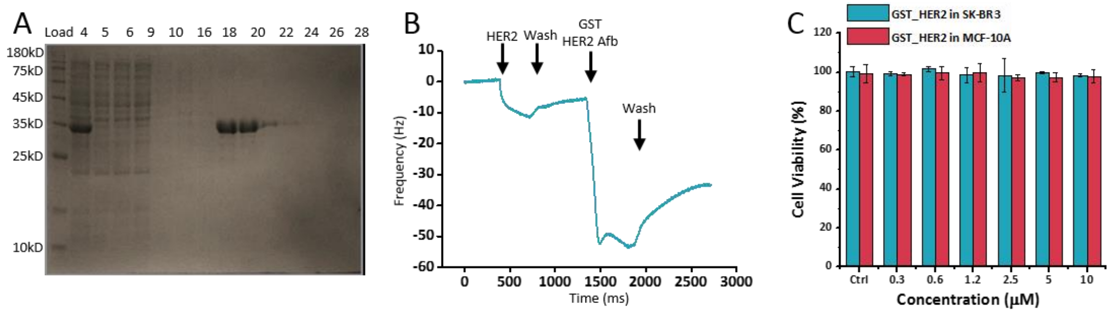

Supplementary Fig. 1. Preparation of GSH-HER2-Afb. (**A**) Purified GST-HER2-Afb identification by SDS-PAGE. (**B**) Quartz crystal microbalance (QCM) analysis of HER2-Afb and GST-HER2-Afb. (**C**) Cell cytotoxicity test of GST-HER2-Afb in SK-BR3 and MCF-10A cells. All bar graphs were reported as means ± standard deviations (SDs) for three experimental replicates (n =3).

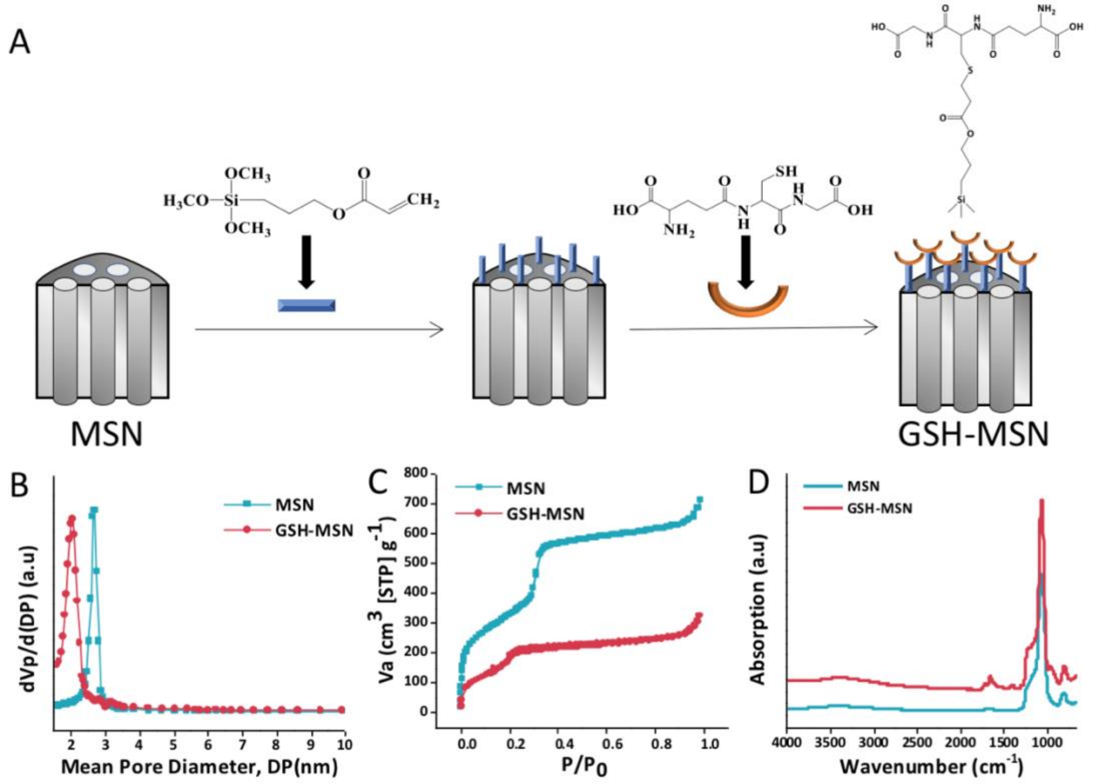

Supplementary Fig. 2. Preparation of GSH-MSN. (**A**) Scheme of GSH-modified mesoporous silica nanoparticles (GSH-MSNs). (**B, C**) Pore size and surface area change from MSNs to GSH-MSNs. (**D**) FTIR analysis for MSNs and GSH-MSNs.

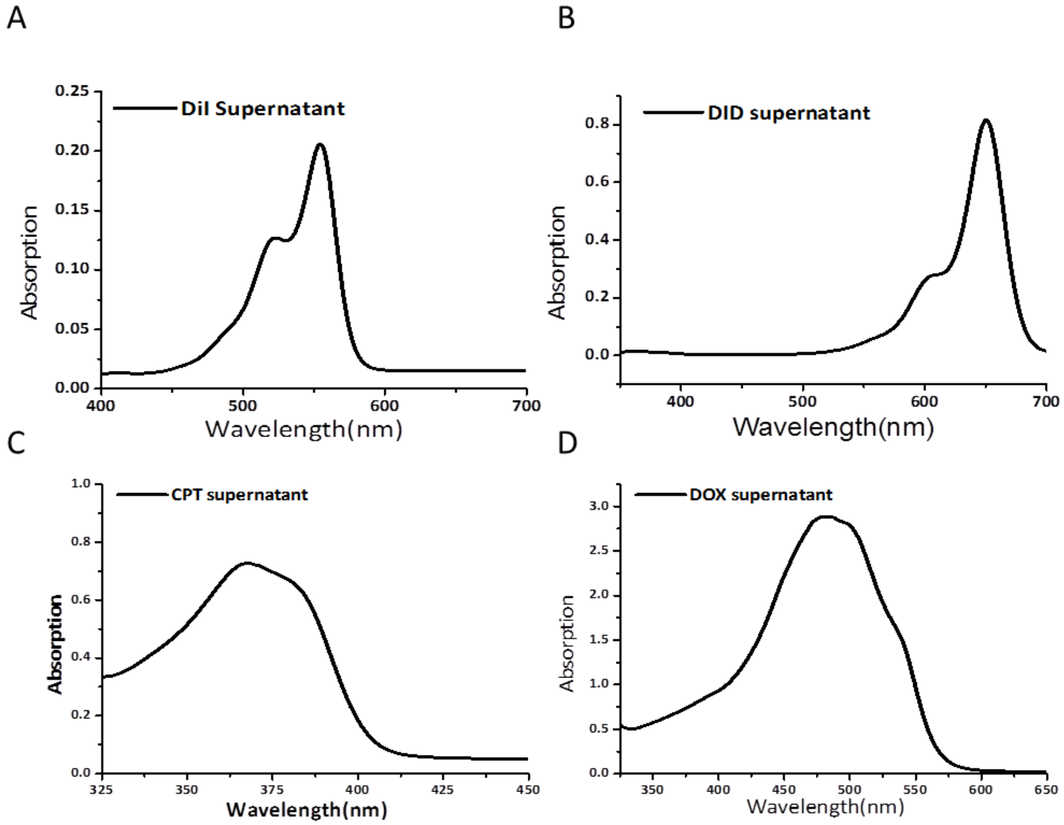

Supplementary Fig. 3. Cargo loading analysis of GSH-MSNs. UV-VIS absorption of the supernatant of (**A**) DiI, (**B**) DiD, (**C**) CPT, and (**D**) DOX solutions.

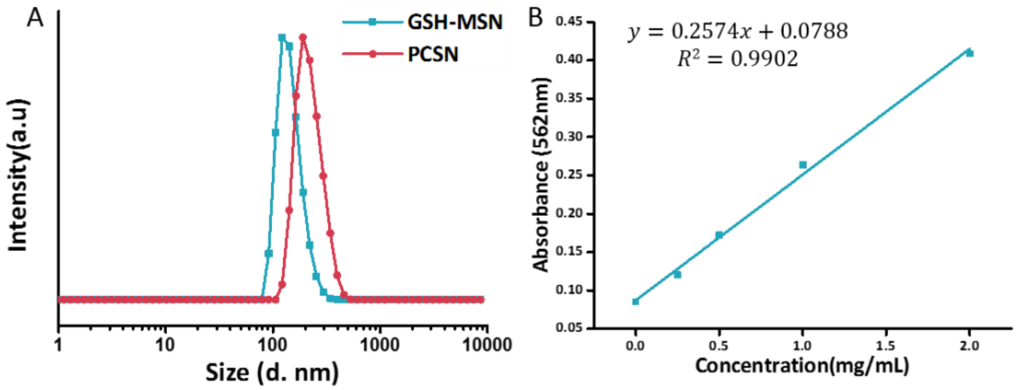

Supplementary Fig. 4. Protein absorption and size difference analysis for PCSNs.(**A**) Hydrodynamics diameter change between GSH-MSNs and PCSNs. (**B**) Concentration curve for BSA on GSH-MSNs determined using the BCA assay. The absorbance of the sample is 0.148, and the concentration is 0.27 mg/mL.

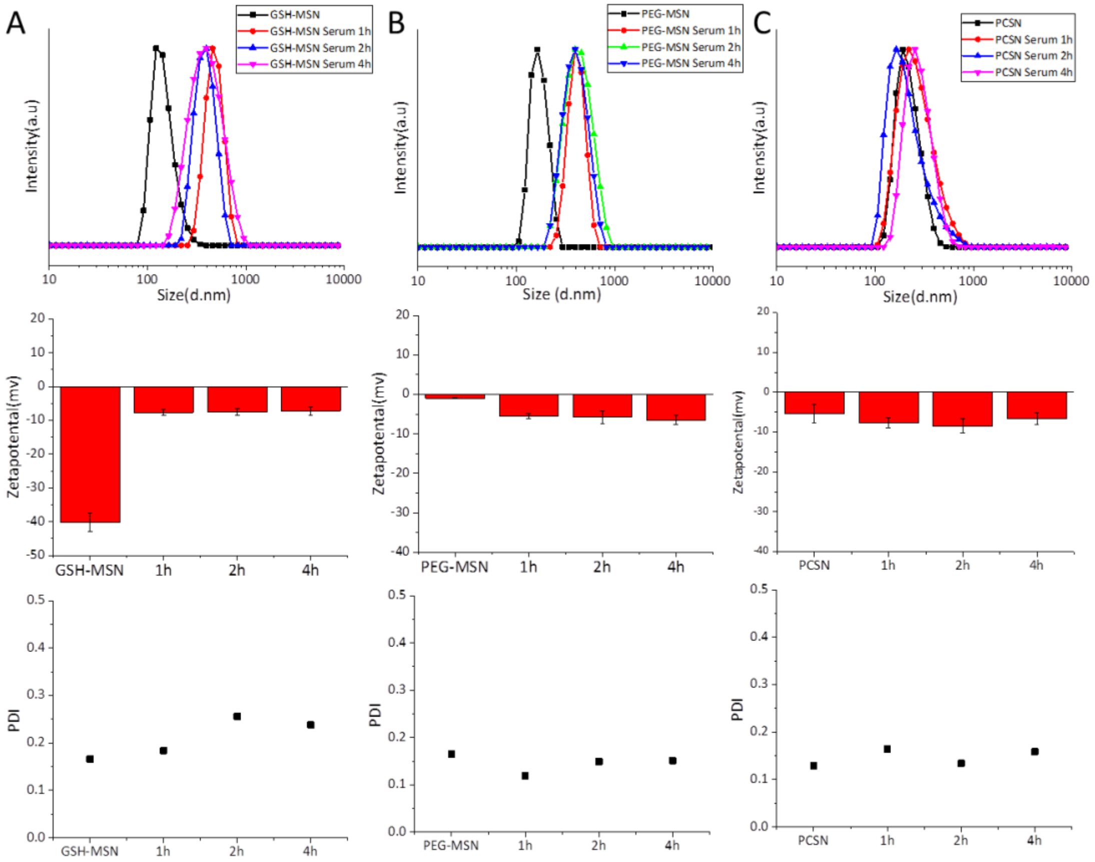

Supplementary Fig. 5. The physical-characterizations of nanoparticle-serum protein interaction. Size, surface charge and PDI analysis of (A) GSH-MSN (B) PEG-MSN (C) PCSN with treatment of the 55% serum for 1h, 2h and 4h.

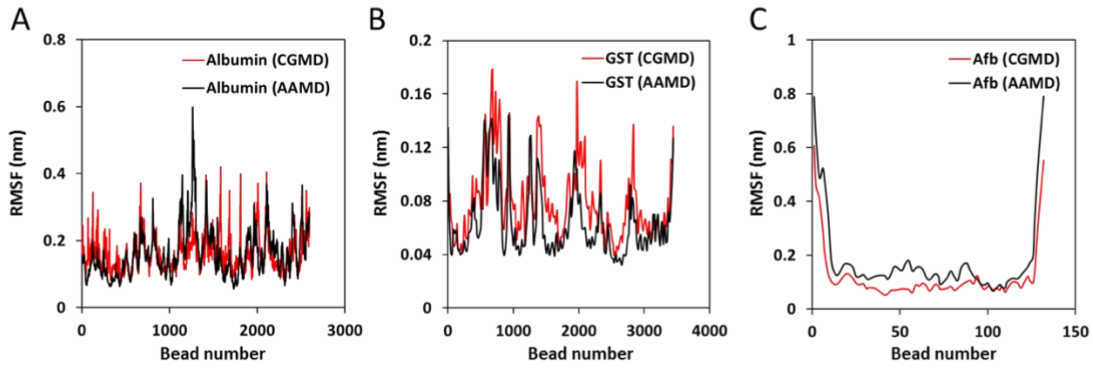

Supplementary Fig. 6. RSMF analysis for CG protein models. RMSF of (**A**) albumin, (**B**) GST and (**C**) Afb.

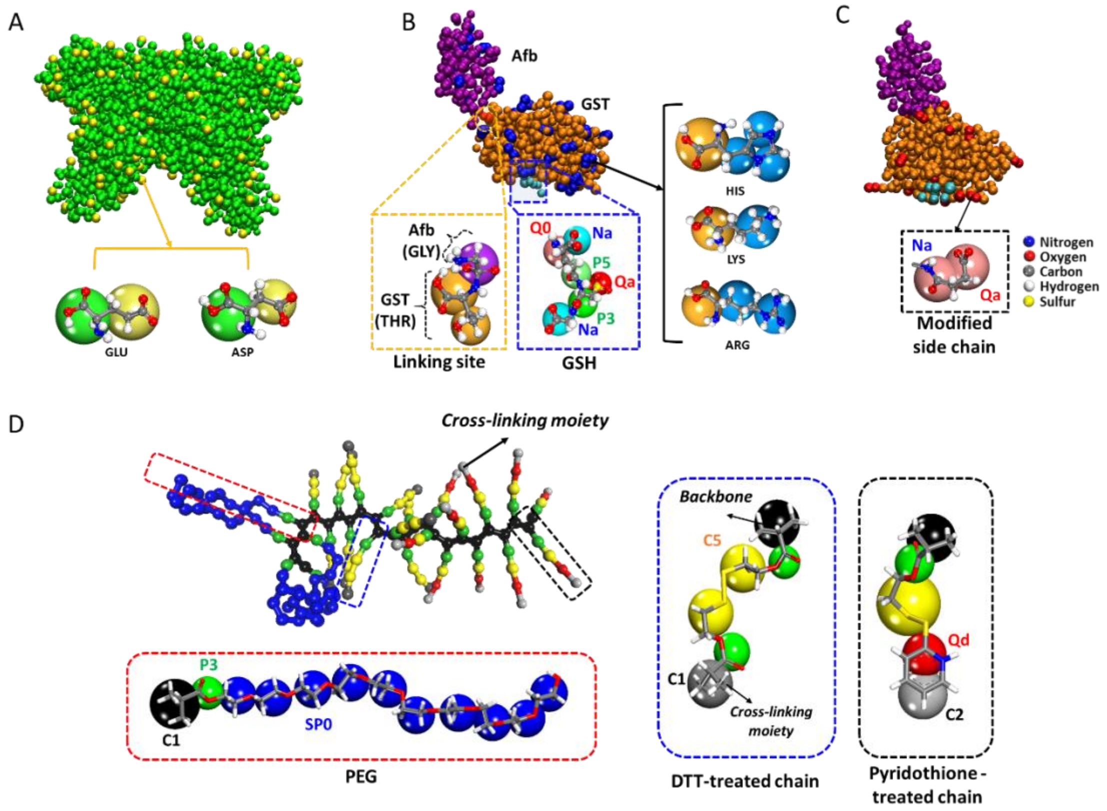

Supplementary Fig. 7. Coarse-grained molecular models. (A) Coarse-grained model of albumin. Negatively charged side chains in GLU and ASP are shown with yellow beads and coarse-grained schemes are presented. Coarse-grained models of **(B)** GST-HER2 Afb and (**C**) charge-modified GST-HER2 Afb. Yellow, blue and black dotted boxes showed magnified coarse-grained mapping of linking site between GST and Afb, GSH and modified side chain in GST, respectively. Positively charged side chains in HIS, LYS and ARG are shown in blue. (**D**) Coarse-grained model of PEG-PDS polymer. Red, blue and black dotted boxes represent PEG chain, DTT-treated chain and pyridothione-treated chain, respectively. Martini bead types for GSH, modified side chain and PEG-PDS polymer were shown with magnification.

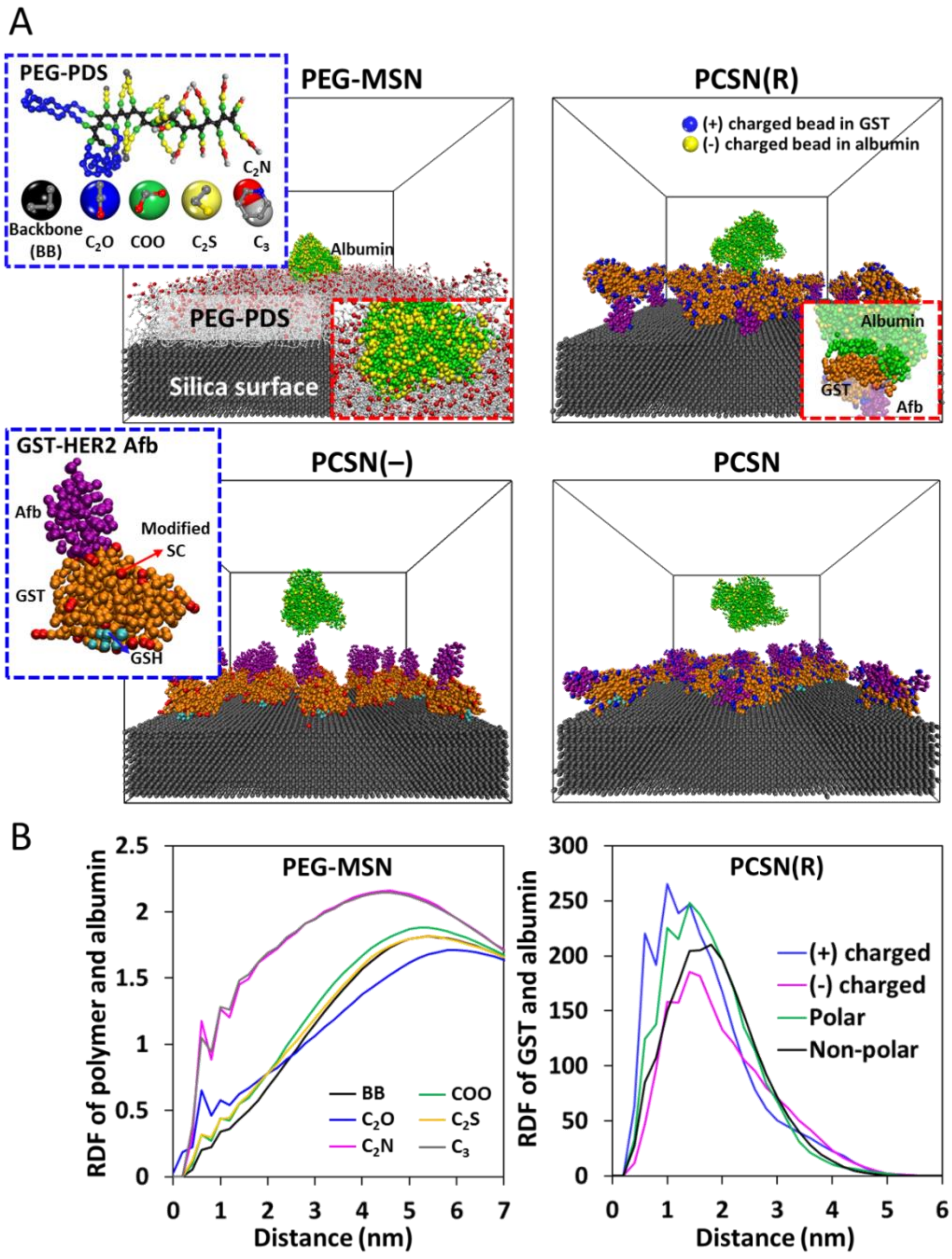

Supplementary Fig. 8. Simulations of surface protein corona phenomenon. (**A**) Final configurations of CGMD simulation of PEG-MSN and PCSN series interacted with albumin in water environment. Water is omitted for clear view. Blue and red dotted boxes show the structures of bead models and binding states, respectively. Color scheme is presented in detail (**Supplementary Fig. 7**) (**B**) Radial distribution functions of constituents of PEG-PDS with albumin in PEG-MSN and residues of GST with albumin in PCSN(R), respectively.

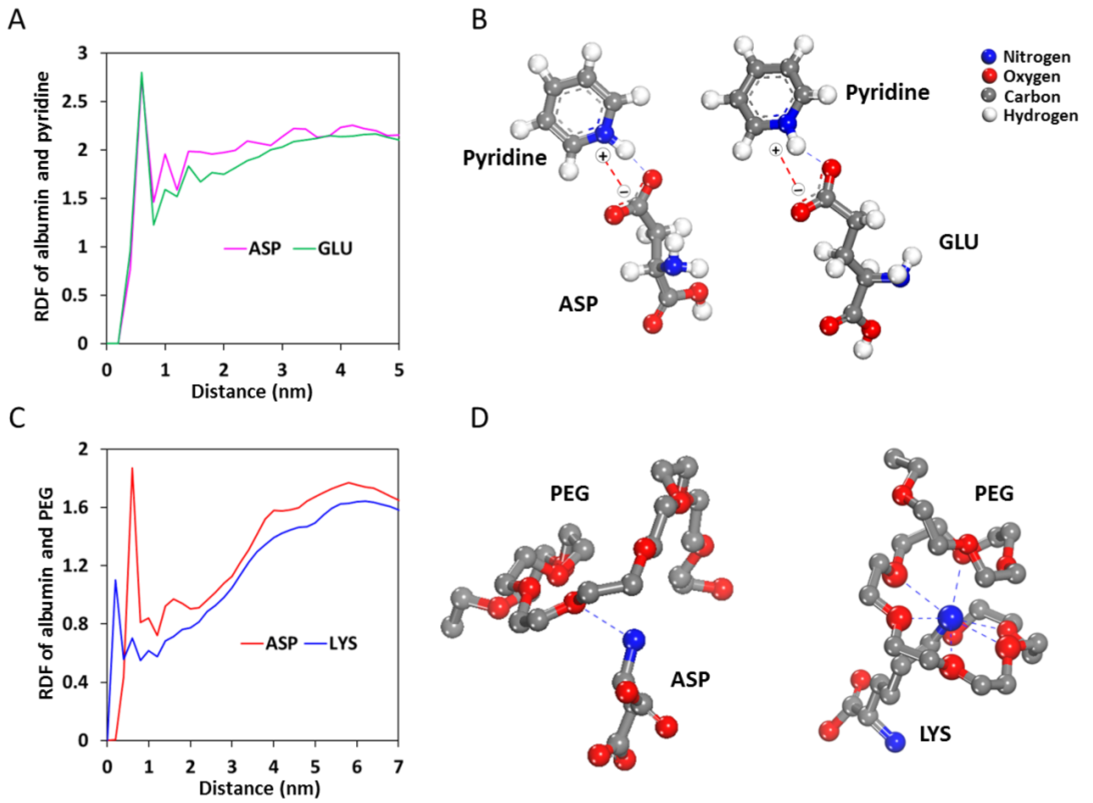

Supplementary Fig. 9. Radial distribution function (RDF) and binding structures in PEG-MSN system. (**A**) RDF of albumin with pyridine in PEG-MSN and **(B)** final configurations of pyridine with ASP and GLU from AAMD simulation. **(C)** RDF of albumin with PEG in PEG-MSN and **(D)** final configurations of PEG with ASP and LYS from AAMD simulation. Residues of albumin were used for RDF analysis. Two RDFs, which were the closest and highest than those of other residues, were shown for clarity. Coulomb interactions, which are presented in red dotted lines, were shown in the pair of pyridine-ASP and pyridine-GLU and hydrogen bonding, which is presented in blue dotted lines, was formed in all the pairs. Also, crown ether-like arrangement of oxygen of PEG with LYS (4) was observed. Hydrogen atoms are not shown in **(D)** for clarity.

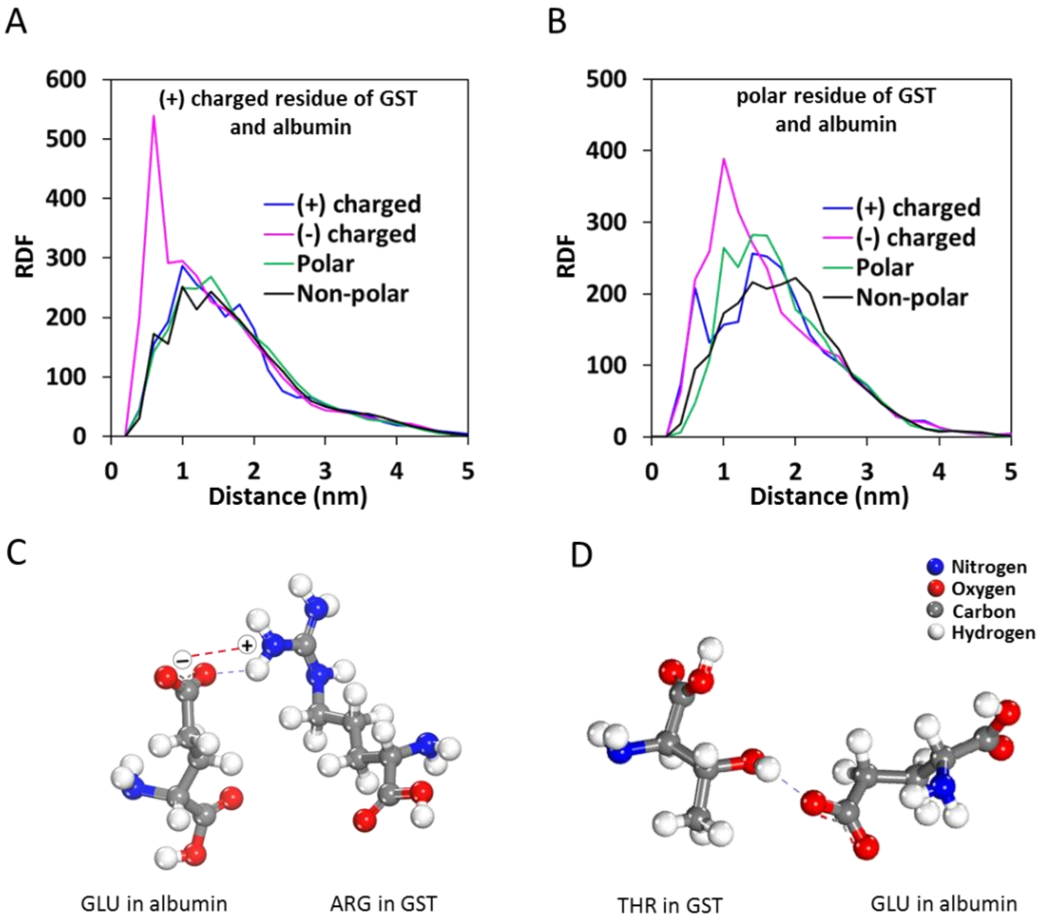

Supplementary Fig. 10. RDF and binding structures in PCSN(R) system. RDFs of albumin with (**A**) positively charged residues of GST and (**B**) polar residues of GST in PCSN(R). Final configurations of (**C**) GLU with ARG and (**D**) THR with GLU from AAMD simulations. Hydrogen-bonding and coulomb interactions, which are presented in blue and red dotted lines, were shown.

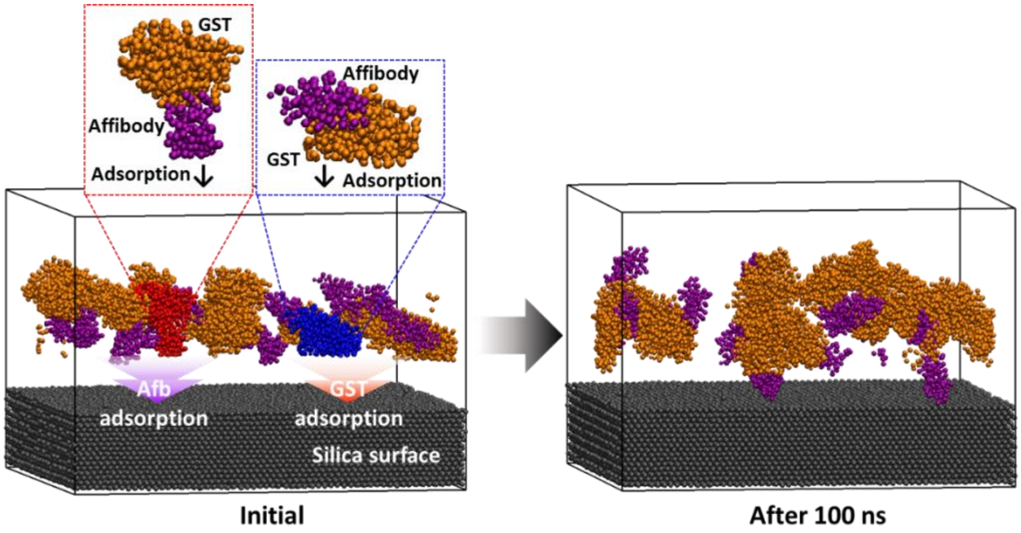

Supplementary Fig. 11. Adsorption of GST-HER2 Afb proteins on silica surface. Initial and final configurations of adsorption simulation with GST-HER2 Afb proteins on silica surface. The red-and blue-highlighted proteins represent models with adsorption directions set to Afb and GST, respectively.

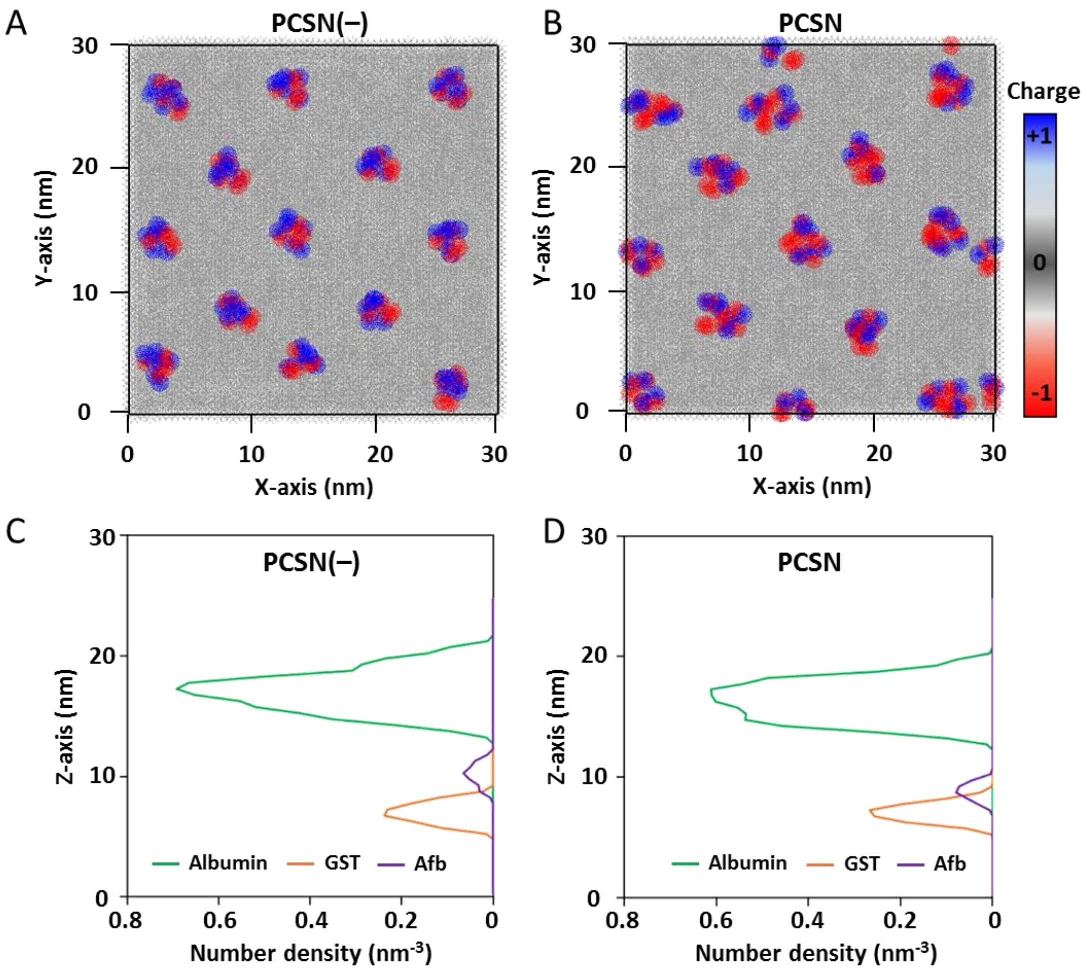

Supplementary Fig. 12. Charge density of Afb and number density along z-axis of simulation system. Charge density of Afb projected on xy-plane in **(A)** PCSN(−) and **(B)** PCSN. Note that PCSN shows wider region of negative charge than PCSN(−). Number density of albumin, GST and Afb along z-axis in **(C)** PCSN(−) and **(D)** PCSN.

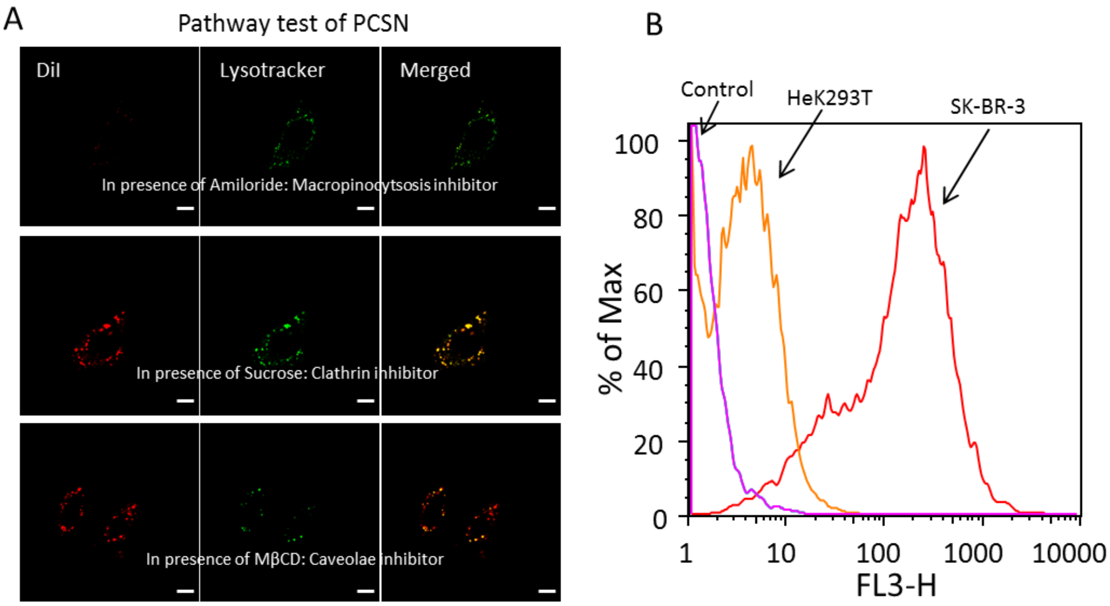

Supplementary Fig. 13. Pathway test of PCSN (**A**) Cellular uptake image of PCSN to SK-BR3 in the presence of amiloride, sucrose , MβCD . (**B**) fluorescence-activated cell sorting analysis of dye (DiI)-loaded PCSN (scale bar is 5 µm).

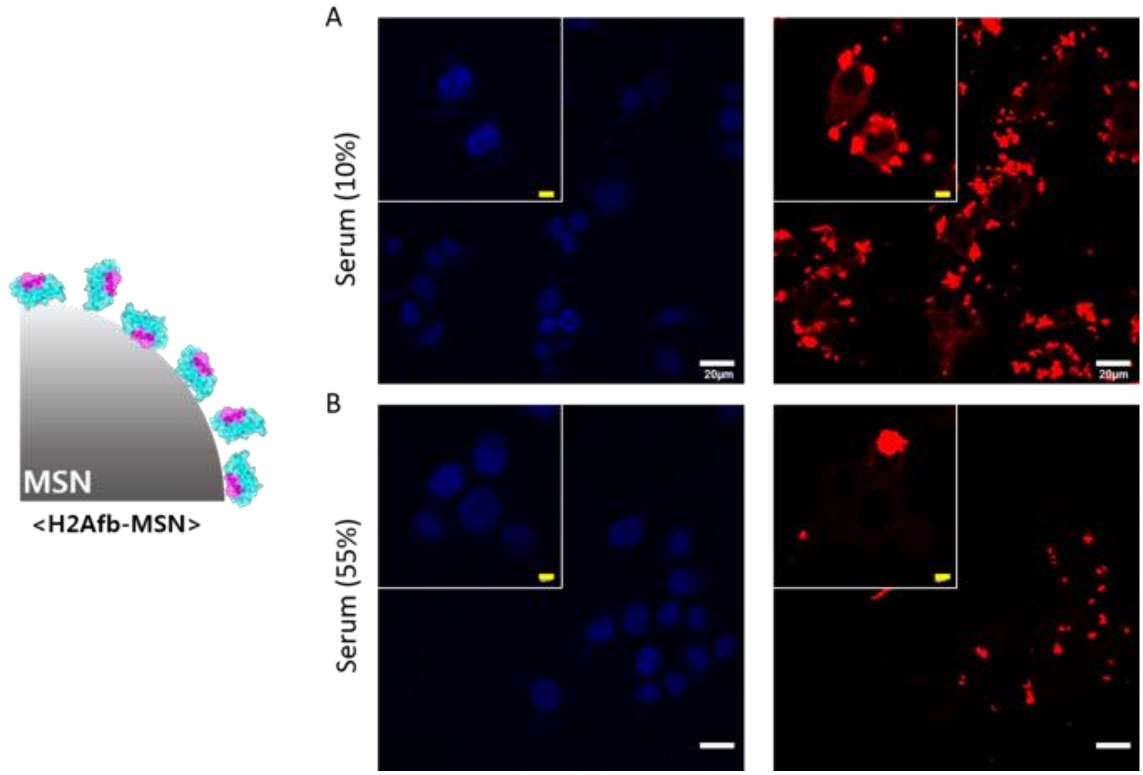

Supplementary Fig. 14. In-vitro experiment for randomly modified MSN. Cellular uptake experiment for dye-containing ($DiIC_{18}$) HER2-binding affibody (Afb) modified MSNs (Afb-MSN) on SK-BR3 cell. The Afb-MSN was pre-treated in **(A)** 10% serum and **(B)** 55% serum for 1 hour (Scale bar is 20 μm and scale bar of inset image is 5 μm).

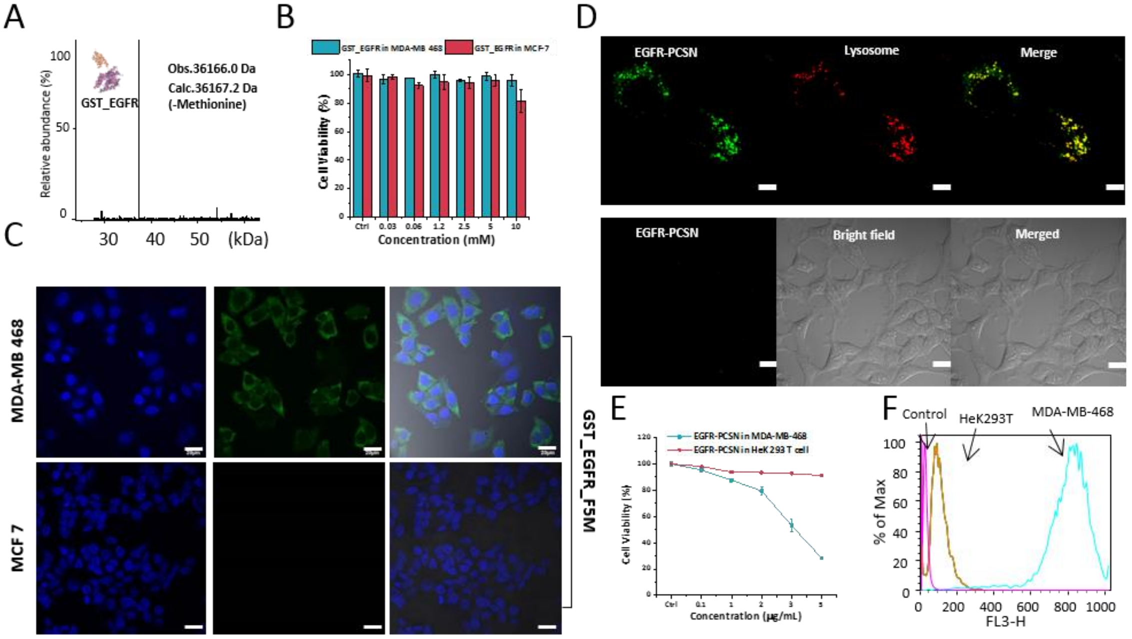

Supplementary Fig. 15. Preparations of GST-EGFR-Afb and EGFR-PCSNs. (A) Mass spectrometry analysis of the GST-EGFR-Afb was 36.1 kDa; (B) cell cytotoxicity test of GST-EGFR-Afb in MDA-MB468 and MCF-10A cells; (C) cellular uptake images of fluorescein 5 maleimide-modified GST-EGFR-Afb (GST-EGFR-Afb F5M) into the target cell (MDA-MB468) and the negative control (MCF-10A) (Scale bar is 20 μm); (D) CPT-loaded EGFR-PCSNs into MDA-MB468 (scale bar is 5 μm) and HeK293T cells (scale bar is 10 μm); (E) cell cytotoxicity assay; (F) fluorescence-activated cell sorting analysis of dye (DiI)-loaded EGFR-PCSNs. Figure B and E graphs were reported as means ± standard deviations (SDs) for three experimental replicates (n =3).

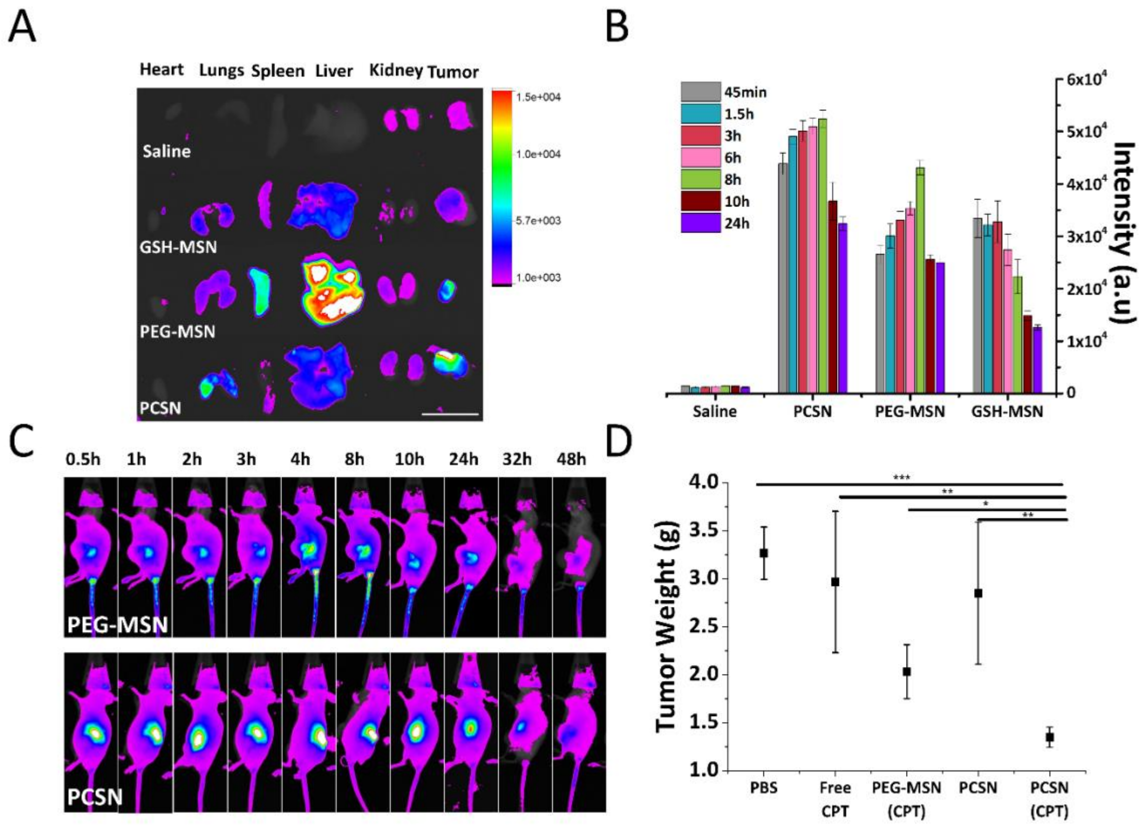

Supplementary Fig. 16. Pre-in vivo and -ex vivo experiments with PCSNs. (A) Ex vivo fluorescence imaging data of each organ; (B) changes in DiD intensity of tumors in each group per each time (each animal received a particle dose of 1 mg/mice (~50 mg/kg), which delivers ~3 mg/kg of DiD per mouse, n = 3/group by IV injection), (Scale bar is 2 cm); (C) representative whole-body fluorescence images of subcutaneous SK-BR3 tumor-bearing mice intravenously injected with nanoparticles (1.5 mg/kg of DiD per mouse, n = 6/group by IV injection); (D) tumor weight of each group (n = 6/group, mean ± SD, statistical significance was calculated by one-way analysis of variance. *P < 0.05, **P < 0.01, *** P < 0.001). All bar graphs were reported as means ± standard deviations (SDs) (n = 6/group).

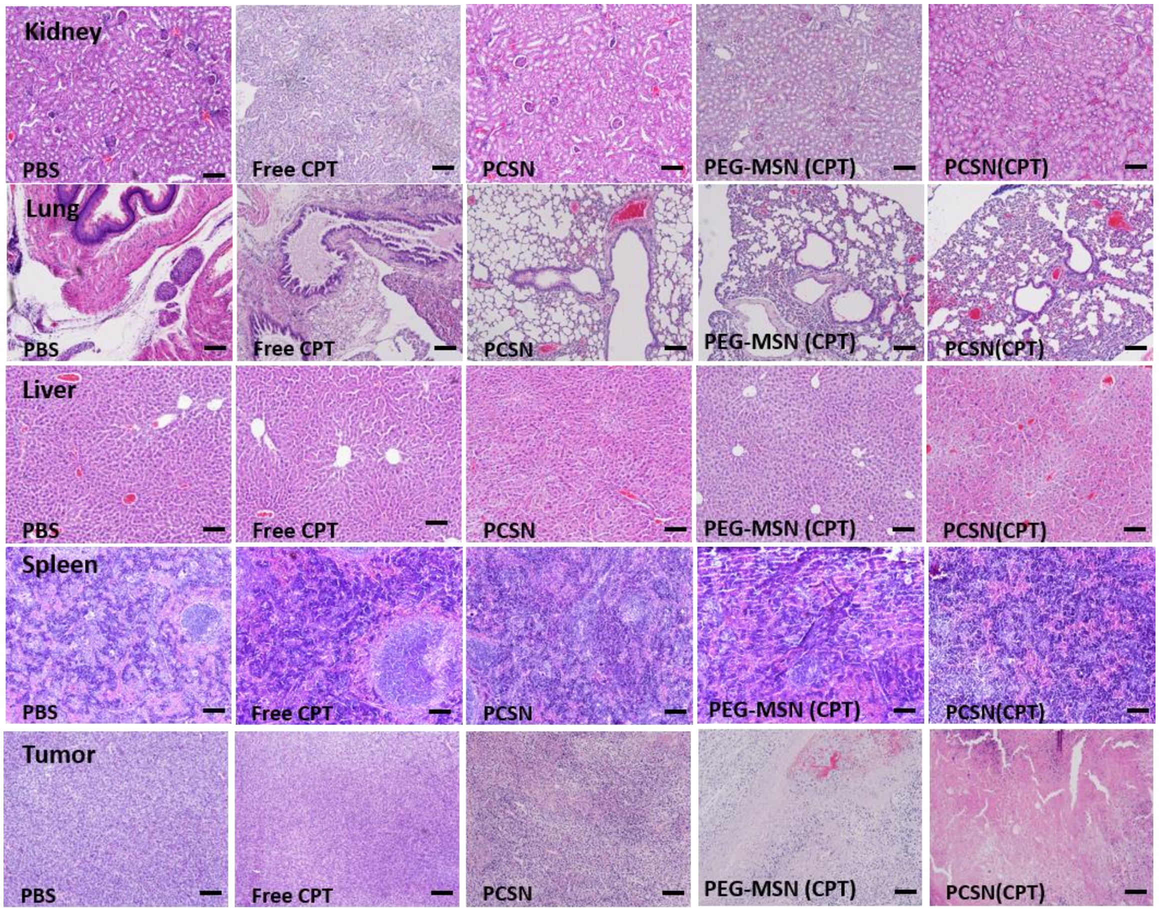

Supplementary Fig. 17. Histological evaluations of tumor tissues. Hematoxylin and eosin (H&E) staining of the lung, liver, spleen, kidney, and tumor. (Scale bar is 50 μm)

Supplementary Tables.

Supplementary Table 1. Difference of surface area, pore volume and pore size between MSN and GSH-MSN

Sample	Surface area (m^2/g)	Pore volume (cm^3/g)	Pore size (nm)
MSN	1190	1.10	2.68
GSH-MSN	540	0.50	2.04

Supplementary Table 2. Ratio of feeding and loading cargo molecule to GSH-MSN

Sample	Feeding ratio	Loading ratio
DOX	200wt%	52%
CPT	200wt%	65%
DiD	50wt%	11%
DiI	20wt%	5%

Supplementary Table 3. Types of amino acids

Type	**Non-polar**	**(+) charge**	**Polar**	**(-) charge**
Residue	Glycine (GLY)<br>Alanine (ALA)<br>Valine(VAL)<br>Proline (PRO)<br>Cysteine (CYS)<br>Leucine (LEU)<br>Isoleucine (ILE)<br>Methionine (MET)<br>Tryptophan (TRY)<br>Phenylalanine (PHE)	Lysine (LYS)<br>Arginine (ARG)<br>Histidine (HIS)	Serine (SER)<br>Threonine (THR)<br>Tyrosine (TYR)<br>Asparagine (ASN)<br>Glutamine (GLN)	Aspartic Acid (ASP)<br>Glutamic Acid (GLU)

Supplementary Table 4. Solvents accessible surface area (SASA) of GST and Afb in PCSN system.

Type	GST (%)	Afb (%)
(+) charged	16.76	17.36
(−) charged	14.90	16.17
Polar	29.38	36.65
Non-polar	38.96	29.82

Supplementary Table 5. Number of residues in GST and Afb

Type	GST	Afb
(+) charged	22	7
(−) charged	27	9
Polar	60	20
Non-polar	105	26

Supplementary Methods.

Preparation of MSN (~100 nm): One gram of CTAB was dissolved in 480 g of 0.015 M NaOH solution and stirred at 80°C. After complete dissolution of surfactant, 4.7 g of TEOS was added, and the mixture was continuously stirred for 2 h at a speed of 7870xg (GYROZEN 1580M, GRF-100-6). The white solid product was filtered through a vacuum filter, washed with DI water, and dried at 70°C in air. The dried product was ground using an agate mortar and finally calcined at 550°C for 5 h.

Preparation of MSN (~50 nm): In total, 1.53 g of CTAB and 0.3 g of TEAH were dissolved in 100 g of DI water. The solutions were stirred at 80°C for 1 h. After complete dissolution of surfactant, 14.45 g of TEOS was added, and the mixture was continuously stirred for 2 h at a speed of 9588xg (LABOGENE 2236R, GRF-L-250-6 Lid). The white solid product was filtered through a vacuum filter, washed with DI water, and dried at 70°C in air. The dried product was ground using an agate mortar and finally calcined at 550°C for 5 h.

Preparation of PEG-MSN: About 5 mg of dye–drug-loaded MSNs were dispersed in 1 mL of DI water containing 10 mg of PEG–PDS polymer and continuously stirred for a period of 12 h at room temperature. To crosslink the polymer shell, a partial amount of DTT was added (50 mol% against the PDS group) and again stirred for a period of 3 h at RT to allow in situ crosslinking. Then, the polymer-capped cargo-loaded MSNs were collected by centrifugation and washed three times with phosphate buffer (pH 7.4; 10 mM) and DI water. The supernatant containing the by product pyridothione released via polymer crosslinking in the thiol-disulfide exchange reaction and any removed drugs were measured using ultraviolet-visible (UV-VIS) spectrometry.

Primers and amino acids sequence.

GST-HER2-Afb

(DNA sequence)

atgggcagcagccatcaccatcatcaccacagccaggatccgatgtcccctatactaggttattggaaaattaagggccttgtgc
aacccactcgacttcttttggaatatcttgaagaaaaatatgaagagcatttgtatgagcgcgatgaaggtgataaatggcgaaac
aaaaagtttgaattgggtttggagtttcccaatcttccttattatattgatggtgatgttaaattaacacagtctatggccatcatacgtta
tatagctgacaagcacaacatgttgggtggttgtccaaaagagcgtgcagagatttcaatgcttgaaggagcggttttggatatta
gatacggtgtttcgagaattgcatatagtaaagactttgaaactctcaaagttgattttcttagcaagctacctgaaatgctgaaaatg
ttcgaagatcgtttatgtcataaaacatatttaaatggtgatcatgtaacccatcctgacttcatgttgtatgacgctcttgatgttgtttt
atacatggacccaatgtgcctggatgcgttcccaaaattagtttgttttaaaaaacgtattgaagctatcccacaaattgataagtact
tgaaatccagcaagtatatagcatggcctttgcagggctggcaagccacgtttggtggtggcgaccatcctccaaaatcggataa
gaattcgggtggtggcttagttccgcgtggttccggaggtggttgcggcggtggcaccggcggtggaagtggaggtggtgcta
gcgtagataacaaatttaataaagaaatgcgaaacgcgtattgggaaatcgccctgttacctaacttaaacaatcagcagaaacg
cgcgtttatcagaagtctctacgatgatccatcacaaagcgcgaatttactggcagaagcaaagaaactcaatgatgctcaggcc
ccaaaagttgat

(Amino acid sequence)
MGSSHHHHHHSQDPMSPILGYWKIKGLVQPTRLLLEYLEEKYEEHLYERDEGDK
WRNKKFELGLEFPNLPYYIDGDVKLTQSMAIIRYIADKHNMLGGCPKERAEISML
EGAVLDIRYGVSRIAYSKDFETLKVDFLSKLPEMLKMFEDRLCHKTYLNGDHVT
HPDFMLYDALDVVLYMDPMCLDAFPKLVCFKKRIEAIPQIDKYLKSSKYIAWPL
QGWQATFGGGDHPPKSDKNSGGGLVPRGSGGGCGGGTGGGSGGGASVDNKFN
KEMRNAYWEIALLPNLNNQQKRAFIRSLYDDPSQSANLLAEAKKLNDAQAPKV
D
(Primer sequence)
HER2Afb_For_NheI
agctagcgtagataacaaatttaataaagaaatgcgaaacgcg
HER2Afb_Rev_SalI
aaagtcgacttaatcaacttttggggcctgagcatc

GST-EGFR-Afb
(DNA sequence)
atgggcagcagccatcaccatcatcaccacagccaggatccgatgtcccctatactaggttattggaaaattaagggccttgtgc
aacccactcgacttcttttggaatatcttgaagaaaaatatgaagagcatttgtatgagcgcgatgaaggtgataaatggcgaaac
aaaaagtttgaattgggtttggagtttcccaatcttccttattatattgatggtgatgttaaattaacacagtctatggccatcatacgtta
tatagctgacaagcacaacatgttgggtggttgtccaaaagagcgtgcagagatttcaatgcttgaaggagcggttttggatatta
gatacggtgtttcgagaattgcatatagtaaagactttgaaactctcaaagttgattttcttagcaagctacctgaaatgctgaaaatg
ttcgaagatcgtttatgtcataaaacatatttaaatggtgatcatgtaacccatcctgacttcatgttgtatgacgctcttgatgttgtttt
atacatggacccaatgtgcctggatgcgttcccaaaattagtttgttttaaaaaacgtattgaagctatcccacaaattgataagtact
tgaaatccagcaagtatatagcatggcctttgcagggctggcaagccacgtttggtggtggcgaccatcctccaaaatcggataa
gaattcgggtggtggcttagttccgcgtggttccggaggtggttgcggcggtggcaccggcggtggaagtggaggtggtgcta
gcgttgataataaattcaacaaagagatgtgggccgcatgggaggaaatccgcaatttaccgaatcttaatggctggcaaatgac
agcttttatagcgtccctggtagatgatccaagccaatctgccaatttacttgcagaggctaaaaagttgaacgacgcccaggcgc
ctaaggtagat

(Amino acid sequence)
MGSSHHHHHHSQDPMSPILGYWKIKGLVQPTRLLLEYLEEKYEEHLYERDEGDK
WRNKKFELGLEFPNLPYYIDGDVKLTQSMAIIRYIADKHNMLGGCPKERAEISML
EGAVLDIRYGVSRIAYSKDFETLKVDFLSKLPEMLKMFEDRLCHKTYLNGDHVT
HPDFMLYDALDVVLYMDPMCLDAFPKLVCFKKRIEAIPQIDKYLKSSKYIAWPL
QGWQATFGGGDHPPKSDKNSGGGLVPRGSGGGCGGGTGGGSGGGASVDNKFN
KEMWAAWEEIRNLPNLNGWQMTAFIASLVDDPSQSANLLAEAKKLNDAQAPK
VD

(Primer sequence)
EGFRAfb_For_NheI
Agctagcgttgataataaattcaacaaagagatgtgggcc
EGFRAfb_Rev_SalI
aaagtcgacttaatctaccttaggcgcctgggcg

Preparation of GST-HER2-Afb and GST-EGFR-Afb: In the genetic modification and purification of GST-HER2-Afb and GST-EGFR-Afb, each HER2 and EGFR affibody-en coding gene with an extra linker (GGGLVPRGSGGGCGGGGTGGGSGGG) was inserte d into an IPTG-induced pETDuet plasmid vector. The plasmid vector contained a hexahis tidine tag and GST-encoding genes in the N-terminus.

The modified gene was amplified by using PCR and confirmed by DNA sequencing. The amplified DNA was transformed to a competent E. coli strain, BL21 (DE), for GST-Afb e xpression. GST-HER2-Afb and GST-EGFR-Afb were overexpressed in LB media for 16 h at 30°C. The E. coli cells were centrifuged at 5000 g for 10 min at 4°C, and the pellets were resuspended in phosphate buffer (50 mM sodium phosphate and 100 mM sodium ch loride, pH 6.5). The suspension was incubated with lysozyme for 20 min at room tempera ture and sonicated for 10 min with 30-s intervals between each incubation. The solution w as centrifuged at 12000 g for 1 h at 4°C. The supernatant was purified by immobilized me tal affinity chromatography (1 mL HisTrap FF column, GE HealthCare) using FPLC. The purified GST-HER2-Afb and GST-EGFR-Afb were dialyzed in PBS (pH 7.4) overnight.

Preparation of GSH-MSN: A total of 100 mg of MSN and 1 mL of 3-(trimethoxysilyl) propyl acrylate were dissolved/mixed in 18 mL of toluene. The solution was stirred at 60°C for 24 h and then washed with ethanol and water. MSN dissolved in 16 mL of DMF was combined with 100 mg of glutathione dissolved in 2 mL of DI water and then vigorously stirred for 72 h at room temperature in the presence of 40 μL of pyridine. After the reaction, MSN was washed with ethanol three times and dried under vacuum at room temperature.

Preparation of PCSN: One milligram of GST-HER2-Afb (or EGFR-Afb) was dissolved in 2 mL PBS (pH 7.4) and slowly added to 3 mL of PBS. Then, 1 mg of GSH-MSN was added and dissolved, and the mixture was stirred for 1 h at 4°C. After centrifuging at 2300xg (Eppendorf Centrifuge 5415R) to remove unreacted protein, PCSN was washed with PBS three times and re-dispersed in 5 mL of PBS.

Preparation of PCSN(R): A mixture of 100 mg of MSN and 1 mL of (3-aminopropyl) trimethoxysilane was mixed in 15 mL of toluene and stirred at 60°C for 24 h. After washing and centrifuging with ethanol and water, MSN was added to a mixture of 50 mg of amine grafted MSN in 9 mL of PBS and 5 mg of disuccinimidyl suberate (DSS) in 1 mL of DMF. This mixture was stirred for 1 h at 4°C. After centrifuging at 2300xg (Eppendorf Centrifuge 5415R) and washing with PBS buffer two times to remove unreacted DSS, MSN was re-dispersed in 3 mL of PBS buffer and slowly added to the GST-HER2-Afb solution (1 mg/2 mL PBS). After stirring for 1 h at 4°C, the mixture was centrifuged at 2300xg (Eppendorf Centrifuge 5415R) to remove unreacted protein, washed with PBS, and PCSN was re-dispersed in 5 mL of PBS.

Preparation of PCSN(-): Succinic anhydride dissolved in 0.1 M 1,4-dioxane was added in small portions to 1 mL of 30 μM GST-HER2-Afb solution in PBS (7.4) buffer for 20 min, and succinic anhydride-modified GST-HER2-Afb was purified by dialysis using PBS buffer for 6 h. GSH-MSN (1 mg/3 mL of PBS) was slowly added to a solution of succinic anhydride-modified GST-HER2-Afb (1 mg/2 mL PBS) and further stirred for 1 h at 4°C.

After centrifuging (2300xg (Eppendorf Centrifuge 5415R)) and washing with PBS three times, PCSN(-) was purified and re-dispersed in 5 mL of PBS for use.

Protein analysis (Proteomcis) LC-MS/MS

After staining with colloidal Coomassie blue, the protein gels were sliced in six consecutive portions followed by in-gel tryptic digestion, as described by Shevchenko and co-workers (1). The resulting tryptic peptides were analyzed by LC-MS/MS. All mass analyses were performed on a Orbitrap ELITE (Thermo, Bremen, Germany) equipped with a nanoelectrospray ion source. To separate the peptide mixture, we used a C18 reverse-phase HPLC column (500 mm × 75 μm ID) using an acetonitrile/0.1% formic acid gradient from 2.4 to 28% for 150 min at a flow rate of 300 nL/ min. For MS/MS analysis, the precursor ion scan MS spectra (m/z 350~1600) were acquired in the Orbitrap at a resolution of 60,000 at m/z 400 with an internal lock mass. The 20 most intensive ions were isolated and fragmented in the linear ion trap by collisionally induced dissociation (CID).

LC-MS/MS data processing

All MS/MS spectra were analyzed using Sequest (Thermo Fisher Scientific, San Jose, CA, USA; Version 1.4.1.14) and X! Tandem (The GPM, thegpm.org; version CYCLONE (2010.12.01.1)). Both programs were set up to search the Bos taurus protein sequence database (8244 entries, UniProt (http://www.uniprot. org/)), which includes frequently observed contaminants, assuming the digestion enzyme trypsin. X! Tandem was set up to search a reverse concatenated subset of the same database also assuming trypsin. Sequest and X! Tandem were searched with a fragment ion mass tolerance of 1.00 Da and a parent ion tolerance of 50.0 ppm. Carbamidomethylation of cysteine and oxidation of methionine were specified as fixed modification and as variable modification, respectively. Oxidation of methionine were specified in Sequest and X! Tandem as variable modifications. Additionally, Glu- >pyro-Glu at the N-terminus, ammonia loss of the N-terminus, and gln->pyro-Glu at the N-terminus were also specified in X! Tandem as variable modifications. Scaffold (Version 4.4.3, Proteome Software Inc., Portland, OR) was used to validate MS/MS-based peptide and protein identifications. Peptide identifications were accepted if they could be established at greater than 99.0% probability to achieve a false discovery rate (FDR) less than 1.0% by the Scaffold Local FDR algorithm. Protein identifications were accepted if they could be established at greater than 99.0% probability to achieve an FDR less than 1.0% and contained at least two identified peptides. Protein probabilities were assigned by the Protein Prophet algorithm (2). Proteins that contained similar peptides and could not be differentiated based on MS/MS analysis alone were grouped to satisfy the principles of parsimony. Proteins were annotated with GO terms from NCBI (downloaded Jun 25, 2017) (3).

Supplementary Notes.

Supplementary Fig. 1: To confirm the interaction between GST-HER2-Afb and human HER2/ErbB 2 protein, we used a quartz crystal microbalance (QCM; Qsense). Human HER2/ErbB 2 protein (Sino Biological) was placed on the gold QCM sensor (Qsense), and unbound HER2/ErbB 2 protein was washed out with PBS buffer. The frequency of the HER2/ErbB 2 protein monolayered QCM sensor decreased with the addition of GST-HER2-Afb.

Supplementary Fig. 2: The FTIR spectra (Fig. S2D) of GSH modified on the mesoporous silica nanoparticles (GSH-MSNs) were characterized by the absorption broad bands near 1711–1651 cm^{-1} (symmetric $vCOO^{-}$), 1409 cm^{-1} (asymmetric $vCOO^{-}$), 1711 cm^{-1} (vC=O) associated with the –COOH group in GSH. Especially, the disappearance in the S–H group (2526 cm^{-1}) in GSH is a clear result of a covalent bond between the acrylate group and GSH. As described in the main text, the surface area and pore size of conventional MSNs tended to decrease by almost half because of modification of GSH.

Supplementary Fig. 3: GSH-MSNs (5.0 mg) were dispersed well in 1 mL of drug solution [10 mg of drug molecules (CPT and DOX) and 1 and 2.5 mg of dye molecules (DiI, DiD), respectively in 1 mL DMSO] and stirred for a period of 12 h at room temperature. After stirring, the drug-loaded GSH-MSNs were centrifuged and the supernatant was collected. The supernatant sample was used to measure drug loading by collecting UV-VIS absorption spectra using a molar absorption coefficient of 42,282 $(M{\cdot}cm)^{-1}$ at λ_{max} = 365 nm (CPT), 11,500 $(M\ cm)^{-1}$ at 478 nm (DOX), 260,000 $(M{\cdot}cm)^{-1}$ at 644 nm (DiD), and 144,000 $(M{\cdot}cm)^{-1}$ at 549 nm (DiI).[4] Drug loading was calculated using the following equation:

Drug loading capacity (%) = Mass of drug in GSH-MSNs/Mass of GSH-MSNs × 100
Entrapment efficiency (%) = Mass of loaded drug/Initial mass of drug × 100

Supplementary Fig. 5: The GSH-MSN, PEG-MSN, and PCSN were treated with 55% serum for 1h, 2h, and 4h, respectively and washed with PBS three times by centrifugation (at 2300xg (Eppendorf Centrifuge 5415R) for 3 minutes). Each nanoparticle was re-dispersed in PBS and measured its size, surface charge, and PDI by Zetasizer Nano ZS. The size of GSH-MSN (140 ± 20 nm) and PEG-MSN (190 ± 20 nm) increased by a factor of 2.5~3, when treated with 55% serum for 1h, 2h, and 4 h, respectively, indicating a protein corona formation. On the other hand, the size of PCSN (~270 ± 20 nm) treated with 55% serum for 1h, 2h, and 4 h was 285 ± 20 nm, 252 ± 20 nm, and 290 ± 20 nm, respectively, showing no significant change. Similarly, the surface charge of nanoparticle was changed from -40 ± 3 mv to -7 ± 1 mv for GSH-MSN and from -1 ± 0.1 mv to -5 ± 1 mv for PEG-MSN when treated with 55% serum, which is presumably due to the screening of the serum proteins. Meanwhile, the change of the surface charge for PCSN was rather negligible. In the case of the PDI, the exposure of the serum did not show significant change for all nanoparticles. All bar graphs were reported as means ± standard deviations (SDs) for three experimental replicates (n =3).

Supplementary Fig. 10: Amino acids are divided into four types, which are positively charged, negatively charged, polar and non-polar types, by the characteristics of side chain as shown in **Supplementary Table 3** (5). RDF of positively charged residue in GST and negatively charged residue in albumin showed the highest peak by ARG in GST and GLU in albumin. Likewise, RDF of polar residue in GST and negatively charged residue in albumin showed the highest peak by THR in GST and GLU in albumin.

Supplementary Fig. 11: GST-HER2 Afb proteins (i.e., 13 ea) were introduced at 4 nm above the silica surface and NVT simulation was performed for 100 ns in water environments. Six and seven of proteins were set to face the silica surface with GST and Afb, respectively, to confirm the adsorption affinity (**Supplementary Fig. 11**). We observed that Afb proteins were mainly adsorbed on the silica surface; Afb location is confirmed in PCSN(R). The affinities of Afb and GST about silica surface were compared by energy calculations, which were averaged with final 50 ns of adsorption simulation. The interaction of Afb was very strong with silica surface (~ -1.43 kJ/mol) while GST was very weak (~ -1.0×10^{-4} kJ/mol). Note that the energy values are theoretically calculated.

Supplementary Fig. 13: To confirm the performance of PCSNs, the dye (DiI) was loaded into PCSNs and incubated with the target cancer cells. As shown in the main text, PCSNs detected only the target cancer cells.We have treated inhibitors of substances interacting with each pathway to determine the exact pathway for the uptake of PCSNs by endocytosis in the target cancer cells. As a result, we confirmed that the macropinocytosis pathway was the main pathway when PCSNs were endocytosed into the cells.

Supplementary Fig. 14: We generated a new HER2-Afb and MSNs was further modified with HER2-Afb through EDC-NHS reaction to give the HER2-Afb-modified MSNs. To investigate the targeting efficacy, dye-containing ($DiIC_{18}$) HER2-Afb-modified MSN was incubated in solutions containing 10% and 55% of serum, respectively, and then treated with SK-BR3 cells. From the confocal microscopy images, we confirmed that cellular uptake for HER2-Afb-modified MSN was reduced and rather stuck outside of the cells, which is presumably due to the screening effect of corona formation on the randomly oriented HER2-Afb of particle (Supplementary Fig. 14). These results suggest that GST plays an important role in reducing interactions among the serum proteins as well as supramolecularly conjugating on particle (Scale bar is 20 μm)

Supplementary Fig. 15: We have synthesized another recombinant protein using protein engineering; the recombinant protein reacted with the EGFR receptor while maintaining the existing GST structure. We hypothesized that if the functional part is changed while maintaining the GST structure, various functions could be applied simultaneously using the PCS structure. The resulting GST-EGFR-Afb had a size similar to that of GST-HER2-Afb, which confirmed successful targeting of the target tumor cell (MDA-MB468). In addition, as with GST-HER2-Afb, we confirmed that GST-EGFR-Afb could react with GSH-MSNs and become a new functional PCSNs. On the basis of these results, it is

expected that if GST-based functional protein is produced by protein engineering, PCSNs having various functions instead of only a targeting ability can be manufactured.

Supplementary Fig. 17: The H&E-stained section analysis revealed no remarkable changes in cellular abnormalities in the PCSN- and PEG-MS-treated groups. The following results were observed: normal appearance of glomeruli in kidney sections, normal hepatocytes in the liver, and usual white and red pulp in the spleen. In the free-CPT-treated groups, mild degenerative changes and necrotic sections appeared in the kidney and hepatic sections of the tissues. There were no representative differences between the control and PCSN treatments.

Coarse-grained model and simulation system

Coarse-grained molecular dynamics (CGMD) was performed with Martini force field (6) to investigate the interaction of external protein (i.e., albumin) with PEG-MSN and PCSN series, which are PCSN, PCSN(−) and PCSN(R) systems. For CG models of protein, residue-based coarse-graining was carried and Martini bead type, which was developed for use in amino acids, was applied (7). CG structures of proteins were compared to all-atom (AA) models by RMSF analysis as shown in **Supplementary Fig. 6** (see All-atom molecular dynamics (MD) simulation section). Albumin was composed of 20 residues of amino acids (**Supplementary Fig. 7A**) and GST-HER2 Afb protein was constructed with chemical bonding between N-terminal of GST and C-terminal of Afb. In PCSN and PCSN(−), GST was bound to silica surface using glutathione (GSH) (**Supplementary Fig. 7B**). Charge-modified GST-HER2 Afb, in which positively charged ammonium groups were substituted with carboxyl groups, was used in PCSN(−) (**Supplementary Fig. 7C**). For PCSN(R), GST-HER2 Afb was randomly coated on the silica surface without GSH. polyethylene glycol-pyridine disulfide hydrochloride (PEG-PDS) polymer was composed of 25 backbone and side chains, which were 5 PEGs, 10 Dithiothreitol (DTT)-treated chains, and 10 pyridothione-treated chains (**Supplementary Fig. 7D**). Note that ten monomers were used for each PEG chain and all DTT-treated chain contained cross-linking moiety. GSH and PEG-PDS polymer were coarse-grained following the rules of Martini, where heavy atoms (3 to 4) were put into a bead unit, and interaction parameters were taken from literatures (8-9). To obtain a cross-linked PEG-PDS molecule, PEG-PDS polymers (i.e. 200 ea) were introduced in a box of $30 \times 30 \times 3$ nm^3. NVT (i.e., isothermal) simulation was performed for 300 ps and if the distance between cross-linking moiety and backbone (BB) bead was less than 4.3 Å during the last 50 ps, cross-linking was performed. All moieties in DTT-treated chains formed bonds with BB to achieve the 50 % of cross-linking density. Cross-linked PEG-PDS polymer was relaxed with NVT simulation for 50 ps and then coated on silica surface. For cross-linking simulation, LAMMPS was performed (10).

For PCSN series and PEG-MSN, thirteen proteins and one cross-linked PEG-PDS polymer were coated on the silica surface, respectively. One albumin was introduced at 4 nm above

the coated silica surface. The thickness of stable silica (0 0 1) surface (11) was 2.78 nm. Silica bead (SP5 martini bead type) was constructed by following Katiyar et al's work (12). The box size of $30 \times 30 \times 25$ nm^3 was used and NVT simulations were performed for 150 ns. The water environments were applied to all simulation systems and counterions (i.e., sodium) were used for the neutrality of the solution. The RDF analysis was performed with simulation configurations collected from the last 50 ns of each simulation. Solvents accessible surface area (SASA) analysis was done with the last configuration of PCSN. All CGMD simulations were performed at 300 K with time step of 10 fs. To keep the isothermal state, velocity rescaling thermostat was used. The cut-off radius of van der Waals interaction was 1.2 nm and particle mesh Ewald (PME) summation was used for coulomb interactions. For CGMD simulations, the GROMACS 5.0.6 package was used (13).

All-atom molecular dynamics (MD) simulation

All-atom molecular dynamics (AAMD) was performed with CHARMM27 force field (14) to investigate the structure of protein models and binding between amino acids and polymer. To investigate the structures of AA protein models one GST, Afb and albumin were used in the box of $8 \times 8 \times 8$ nm^3, $6 \times 6 \times 6$ nm^3 and $12 \times 12 \times 12$ nm^3, respectively, which were filled with water. NPT (i.e., isothermal-isobaric) simulations for 20 ns were performed and root mean square fluctuation (RMSF) was analyzed with the trajectory of final 10 ns. Note that original protein models of albumin, GST and Afb were obtained from Protein Data Bank (PDB) (15-17). Also, to find the binding structure between amino acids and polymer, NPT simulations for 200 ps were performed in the box of $25 \times 25 \times 25$ nm^3, filled with water. Pairs of pyridine–aspartic acid (ASP), pyridine–glutamic acid (GLU), polyethylene glycol (PEG)–ASP, PEG–lysine (LYS), GLU-ARG, and GLU-THR were used for the simulation.

Summary of the simulation

Albumin was used to investigate the interactions between coated silica nanoparticles and serum protein because it was the most abundantly adsorbed group of serum proteins with coated silica nanoparticles (**Fig. 2E**). As shown in **Supplementary Fig. 8A**, the adsorption of albumin occurred in PEG-MSN and PCSN(R), respectively. From the radial distribution function (RDF) (**Supplementary Fig. 8B**), we found that the binding of albumin on coated polymer (i.e., PEG-PDS) was originated from C_2N and C_3 groups in pyridine and C_2O group in PEG while positively-charged or polar residues of GST in PCSN(R) had good affinity with albumin in PCSN(R). Specifically, the interaction types were identified via molecular structures obtained from all-atom molecular dynamics; coulomb interaction with hydrogen bonding for pyridine and hydrogen bonding for PEG in PEG-MSN (**Supplementary Fig. 9**) and coulomb interaction with hydrogen bonding for positively charged residues of GST and hydrogen bonding for polar residues of GST in PCSN(R) (**Supplementary Fig. 10**). In terms of interaction with silica surface, Afb in GST-HER2

Afb naturally showed strong affinity with the surface (**Supplementary Fig. 11**) while the GST part was attached to the surface due to the GSH linkage. Once Afb was outward from the surface as such in the cases of PCSN(−) and PCSN systems, albumin was not adsorbed. Afb did not form a favorable interaction with albumin because the relative area of exposed negatively-charged particles in Afb was large and the absolute number of polar residues was small (**Supplementary Table S4, S5**). Interestingly, we observed that in PCSN, Afb was slightly laid down with GST (**Supplementary Fig. 8A**) so that the negatively charged particles was further outward (**Supplementary Fig. 12A** and **B**) compared to that in PCSN(−). Note that succinic anhydride-modified side chain is negatively charged so that Afb in PCSN(−) was not laid down but slightly extended outward. This repulsive electrostatic interaction made albumin remained farther than ~ 1.5 nm from PCSN than PCSN(−) (**Supplementary Fig. 12C** and **D**). In the PCSN series, the location (outward or inward) of Afb on the surface turned out to be a very important factor for the nanoparticle's cloaking state.

Supplementary References.

1. Shevchenko, A., Tomas, H., Havlis, J., Olsen, J. V. & Mann, M. In-gel digestion for mass spectrometric characterization of proteins and proteomes. Nat. Protoc. 1, 2856-2860 (2007).

2. Mueller, L. N., Brusniak, M.-Y., Mani, D. & Aebersold, R. An assessment of software solutions for the analysis of mass spectrometry based quantitative proteomics data. J. Proteome Res. 7, 51-61 (2008).

3. Consortium, G. O. Ashburner M, Ball CA, Blake JA, Botstein D, Butler H, Cherry JM, Davis AP, Dolinski K, Dwight SS, Eppig JT, Harris MA, Hill DP, Issel-Tarver L, Kasarskis A, Lewis S, Matese JC, Richardson JE, Ringwald M, Rubin GM, Sherlock G. 2000. Gene ontology: tool for the unification of biology. Nat. Genet. 25, 25-29.

4. Musselman, E. D. *Insights into interactions between poly(ethylene glycol) and proteins from molecular dynamics simulations.* (University of Iowa, 2010)

5. Cooper, G. M. & Hausman, R. E. *The Cell: A Molecular Approach.* (ASM Press, Washington, D.C, 2004), p. 51.

6. Marrink, S. J., Risselada, H. J., Yefimov, S., Tieleman, D. P. & de Vries, A. H. The martini force field: coarse grained model for biomolecular simulations. *J. Phys. Chem. B* **111**, 7812-7824 (2007).

7. Monticelli, L. et al., The MARTINI Coarse-Grained Force Field: Extension to Proteins. *J. Chem. Theory. Comput.* **4**, 819-834 (2008).

8. Zhang, L., Bai, S. & Sun, Y. Modification of Martini force field for molecular dynamics simulation

of hydrophobic charge induction chromatography of lysozyme. *J. Mol. Graph. Model.* **29**, 906-914 (2011).

9. Rossi, G., Fuchs, P. F., Barnoud, J. & Monticelli, L. Coarse-grained MARTINI Model of Polyethylene Glycol and of Polyoxyethylene Alkyl Ether Surfactants. *J. Phys. Chem. B* **116**, 14353-14362 (2012).

10. Plimpton, S. Fast Parallel Algorithms for Short-Range Molecular Dynamics, *J. Comp. Phys.* **117**, 1-19 (1995)

11. Goumans, T. P. M., Wander, A., Brown, W. A. & Catlow, C. R. A. Structure and stability of the (001) a-quartz surface. *Phys. Chem. Chem. Phys*. **9**, 2146–2152 (2007).

12. Katiyar, P. & Singh, J. K. A coarse-grain molecular dynamics study of oil-water interfaces in the presence of silica nanoparticles and nonionic surfactants. *J. Chem. Phys.* **146**, 204702 (2017).

13. Van Der Spoel, D., Lindahl, E., Hess, B., Groenhof, G. & Mark, A. E. Berendsen, GROMACS: fast, flexible, and free. *J. Comput. Chem.* **26**, 1701-1718 (2005).

14. Foloppe, N. & MacKerell, A. D. All-atom empirical force field for nucleic acids: I. Parameter optimization based on small molecule and condensed phase macromolecular target data. *J. Comput. Chem.* **21**, 86-104 (2000)

15. Sugio, S., Kashima, A., Mochizuki, S., Noda, M. & Kobayashi, K. Crystal structure of human serum albumin at 2.5 A resolution. *Protein Eng.* **12**, 439-446 (1999) (PDB ID: 1AO6)

16. Reinemer, P. et al., Three-dimensional structure of glutathione S-transferase from Arabidopsis thaliana at 2.2 A resolution: structural characterization of herbicide-conjugating plant glutathione S-transferases and a novel active site architecture. *J. Mol. Biol.* **255**, 289-309 (1996) (PDB ID: 1GNW)

17. Lindborg, M. et al., High-affinity binding to staphylococcal protein A by an engineered dimeric Affibody molecule, *Protein Eng. Des. Sel*. **26**, 635-644 (2013) (PDB ID: 2M5A)
